# Supplementary material for: Comparison of double chevron-cut and biplanar distal femoral osteotomy techniques: A biomechanical study
Source: PLoS One. 2024 Apr 18;19(4):e0296300. doi: 10.1371/journal.pone.0296300 (PMC11025861; doi:10.1371/journal.pone.0296300)
Supplement: S1 File — (PDF) [file pone.0296300.s001.pdf]

Experimental set-up: Axial Loading

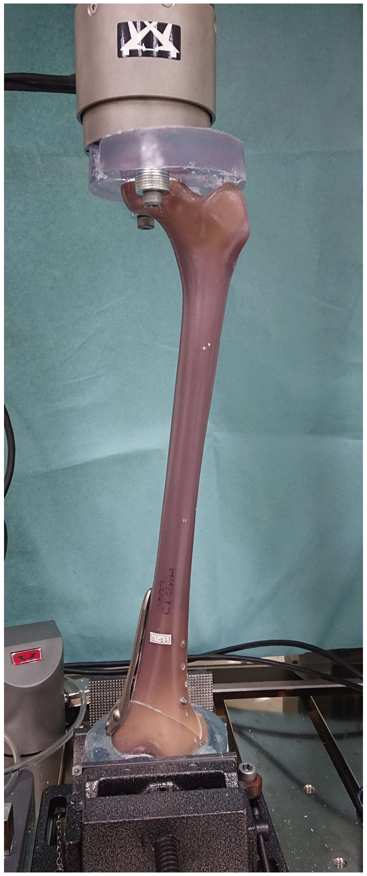

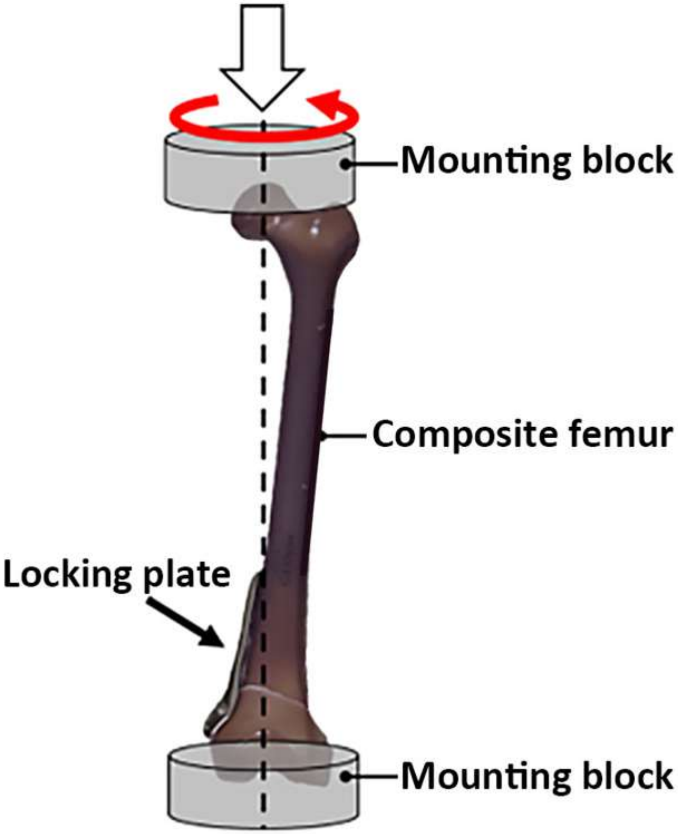

Axial Loading

Test Sequence	Number of cycles	Axial preload (N)	Axial Load (N)	Torsional preload (Nm)	Torsional Load (Nm)
1	100	10	150	0	0
2	100	10	800	0	0
3	1	0	To failure	0	0

Experimental set-up: Torsional Loading

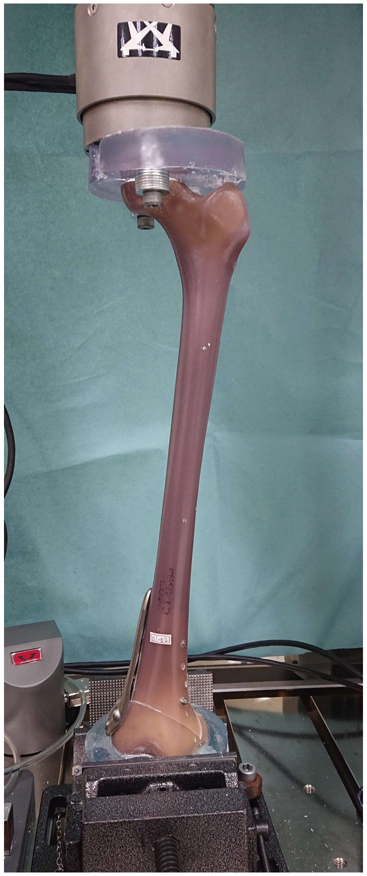

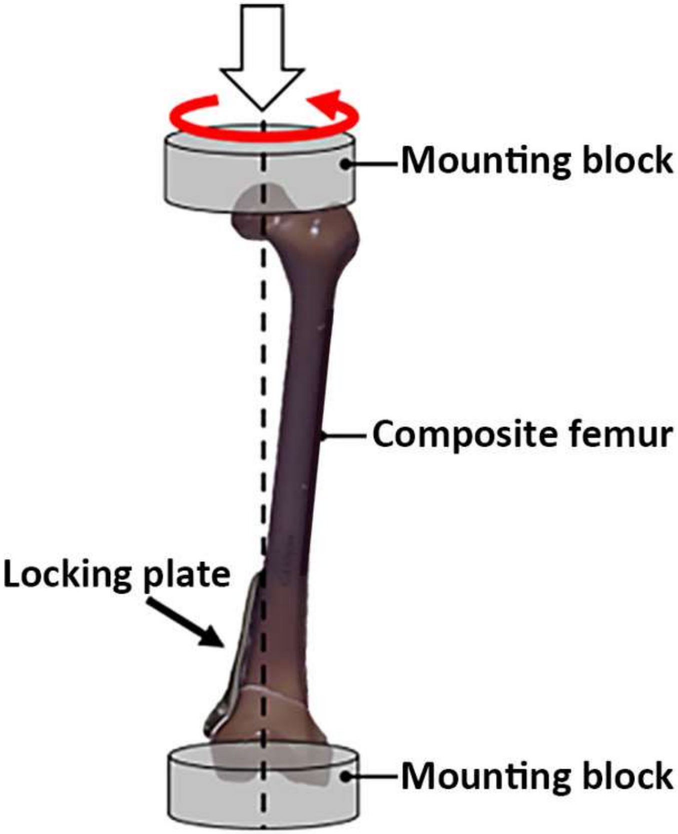

Torsional Loading

Test Sequence	Number of cycles	Axial preload (N)	Axial Load (N)	Torsional preload (Nm)	Torsional Load (Nm)
1	100	0	0	0.5	5
2	100	150	0	0.5	5
3	100	800	0	0.5	5
4	1	0	0	0	To failure

Data Collection for Testing

Compression Test

1-1. Maximum Load Value: The maximum load value measured by the testing machine, in Newtons.

1-2. Maximum Compression Displacement: The maximum compression displacement endured by the sample under the maximum load value, in millimeters.

1-3. System Stiffness: Maximum load value divided by maximum compression displacement, in N/mm.

Torsion Test

2-1. Maximum Torque Value: The maximum torque value measured by the testing machine, in Newton-meters.

2-2. Maximum Torsion Angle: The maximum torsion angle endured by the sample under the maximum torque value, in degrees.

2-3. System Stiffness: Maximum torque value divided by maximum torsion angle, in Nm/degree.

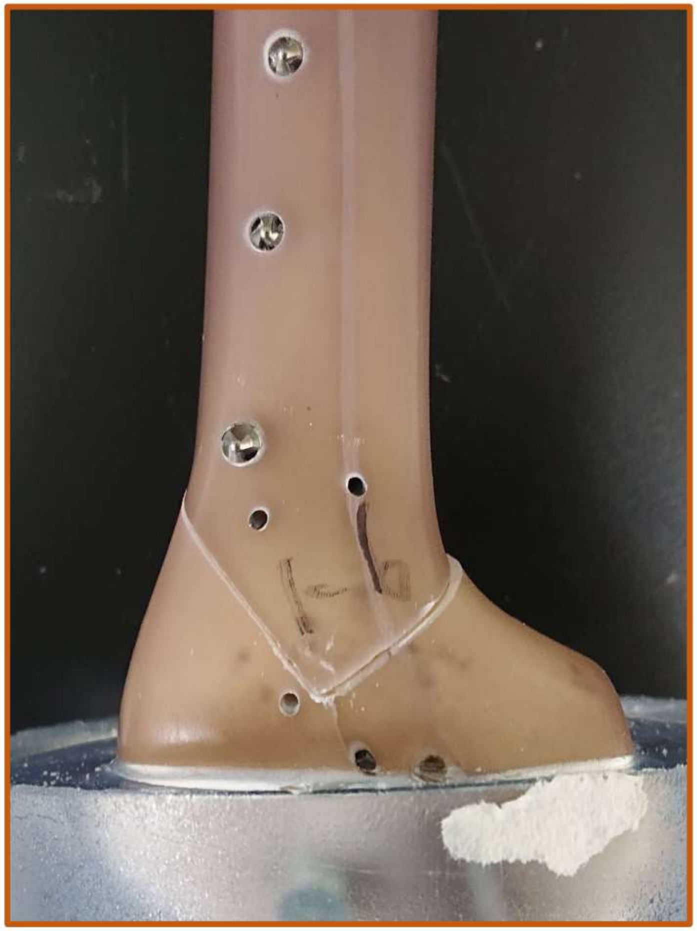

Test Results - Torsion Test

Group	Sample Number	Sequence 1	Sequence2	Sequence3	Sequence4
V type	01-01	Passed the test	Passed the test	Passed the test	Failure
	01-02	Passed the test	Passed the test	Passed the test	Failure
	01-03	Passed the test	Passed the test	Passed the test	Failure
L type	02-01	Passed the test	Passed the test	Passed the test	Failure
	02-02	Passed the test	Passed the test	Passed the test	Failure
	02-03	Passed the test	Passed the test	Passed the test	Failure

Group	Sample Number	Maximum Torque Value (N)	Rotation Angle (mm)	System Stiffness (Nm/degree)
V type	01-01	62.89	3.16	19.90
	01-02	73.63	2.85	25.84
	01-03	52.48	1.49	35.22
L type	02-01	59.48	19.29	3.08
	02-02	66.04	14.57	4.53
	02-03	42.56	7.08	6.01

Test Results - Compression Test

Group	Sample Number	Sequence 1	Sequence2	Sequence3
V type	01-04	Passed the test	Passed the test	Failure
	01-05	Passed the test	Passed the test	Failure
	01-06	Passed the test	Passed the test	Failure
L type	02-04	Passed the test	Passed the test	Failure
	02-05	Passed the test	Passed the test	Failure
	02-06	Passed the test	Passed the test	Failure

Group	Sample Number	Maximum Load Value (N)	Displacement (mm)	System Stiffness (N/mm)
V type	01-04	8153.75	4.83	1688.15
	01-05	7669.06	8.10	946.80
	01-06	8702.23	8.05	1081.02
L type	02-04	8543.32	7.69	1110.97
	02-05	8726.12	6.37	1369.88
	02-06	6228.62	4.61	1351.11
