# Supplementary material for: Comparison of double chevron-cut and biplanar distal femoral osteotomy techniques: A biomechanical study
Source: PLoS One. 2024 Apr 18;19(4):e0296300. doi: 10.1371/journal.pone.0296300 (PMC11025861; doi:10.1371/journal.pone.0296300)
Supplement: Attachment — Submitted filename: Response to reviewers.docx [file pone.0296300.s003.docx]

Dear Reviewers,

Thank you for your detailed feedback on our manuscript, which has greatly improved our manuscript. We have revised the manuscript and tables according to your suggestions. We hope that the revised version meets the standards required for publication in "PLOS ONE." Please find our responses to your comments below:

**Reviewer #1:**

I am of the opinion that the revised version of this paper meets the standards for publication. I would like to propose its consideration for publication in its current revised format.

**Author’s Response:**

Thank you for your positive feedback on our revised manuscript. We appreciate your support for its consideration for publication in its current form. Your insights have been invaluable in improving our work.

We look forward to the possibility of our paper being published in PLOS ONE.

**Reviewer #2:**

In this revised version of the manuscript and the document describing the authors’ response to the reviewer, the authors have appropriately responded to each of the comments given. Consequently, the clarity of the description has been improved; however, there are a few areas requiring further clarification as described below.

**Q1: Lines 112-118**

Please describe in more detail regarding how the specimen was positioned and aligned to mimic the normal weight bearing.

**Author’s Response:**

We appreciate the reviewer's feedback and their request for more detailed information on how the specimen was positioned and aligned to mimic normal weight bearing. In response to this valuable suggestion, we have revised the manuscript to provide a more comprehensive description of the alignment procedure, which we believe will enhance the clarity and understanding of our experimental setup. We hope that these additions address the reviewer's concern adequately. Thank you for your constructive comments. (Line 110-115)

**Q2: Lines 238-251**

It is still not clear enough which aspect of the study results can ensure the feasibility of immediate weight bearing in DCC osteotomy procedure.

**Author’s Response:**

We appreciate the reviewer's feedback. The biomechanical study focused on assessing mechanical stability and stiffness. The connection between the study results and immediate weight bearing in the DCC osteotomy procedure lies in the hypothesis that greater mechanical stability, particularly superior rotational stiffness observed in the DCC group, may enable patients to initiate early weight-bearing based on comfort and pain tolerance. This aspect contributes to the clinical outcome observed in our previous study with a mean time to full weight bearing of 3.7 weeks. We have clarified this connection in the revised manuscript. (Line 244-253)
